# Efficacy of dietary supplementation with 1α-hydroxycholecalciferol on performance, eggshell quality, serum metabolites, jejunal morphology and bone characteristics of laying hens at the late stage of production

**DOI:** 10.1016/j.psj.2024.104618

**Published:** 2024-12-02

**Authors:** Maryam Ghaderi Nik, Reza Mahdavi, Shahab Ghazi, Kourosh Gholami

**Affiliations:** Department of Animal Science, College of Agriculture and Natural Resources, Razi University, Kermanshah, Iran

**Keywords:** 1α-hydroxycholecalciferol, Laying hens, Bone quality, Enzyme activity, Gut morphology

## Abstract

The present study evaluated the effects of 1α-hydroxycholecalciferol (1αOHD_3_) supplementation on performance, egg quality, gut morphology, serum metabolites, and bone characteristics of Lohman LSL-Lite laying hens. A total of 180 birds (110 weeks of age) were allocated according to a completely randomized design with five treatments. Each treatment had six replicates containing six hens each. The treatments consisted of basal diet with 2000 IU/kg vitamin D_3_, basal diet supplemented with 1.5, 3, 4.5, and 6 μg/kg of 1αOHD_3_. Results showed that dietary supplementation with 1αOHD_3_ increased the egg production (linear P = 0.002 and quadratic P = 0.009) and gross revenue (linear P = 0.042) whilst it decreased the abnormal eggs (linear P = 0.004 and quadratic P = 0.009) in aged laying hens. Similarly, it linearly and quadratically increased the shell thickness and eggshell strength (P < 0.001). Egg mass (linear P = 0.075) showed a tendency to increase with increasing dietary 1αOHD_3_ supplementation levels. The egg quality parameters, including Haugh unit, relative weight of albumen, yolk and eggshell were not affected by the treatments (P > 0.05). Furthermore, 1αOHD_3_ supplementation increased the serum levels of calcium (linear P = 0.003 and quadratic P = 0.011), albumin (linear P = 0.016 and quadratic P = 0.033), vitamin D (linear and quadratic P < 0.001), alanine aminotransferase activity (linear P = 0.02) whilst the addition of 1αOHD_3_ decreased alkaline phosphatase activity (linear P = 0.003 and quadratic P = 0.011), without affecting the serum levels of phosphorus and aspartate aminotransferase activity (P > 0.05) in laying hens. In addition, the linear tendency to increase was observed (linear P = 0.062) in total protein. Dietary supplementation of 1αOHD_3_ increased the tibia diameter (linear P = 0.053), tibia calcium (linear P = 0.004 and quadratic P = 0.014) and tibia strength (linear and quadratic P < 0.001). The addition of 1αOHD_3_ did not affect the phosphorus and ash of the tibia (P > 0.05). Linear and quadratic responses were found for crypt depth (linear and quadratic P = 0.001) and villus height to crypt depth ratio (linear P = 0.004 and quadratic P = 0.010). The experimental treatments did not affect the jejunal villus height, villus width and villus surface area in aged laying hens (P > 0.05). Our findings suggest that the inclusion of 1αOHD_3_ is beneficial, as it enhances egg production, profitability, eggshell thickness, and tibia quality while reducing the incidence of abnormal eggs during the later phase of egg production.

## Introduction

The stage of egg production that follows 60 weeks of age is frequently termed the late phase of production. It is characterized by a significant age-related reduction in overall performance, (egg production and FCR) and egg quality (eggshell quality and Haugh unit). This production cycle is typically associated with an increase in reactive oxygen species, lower antioxidant capacity, reduced reproductive hormone secretion, and compromised intestinal oxidative status. Ultimately, this leads to substantial economic losses and poor eggshell quality, raising concerns among producers (Park & Sohn, 2018; Yu et al., 2023). The enhancement of production performance and egg quality in laying hens during the late laying period has become a critical issue for researchers and poultry producers. Improving the production performance and eggshell quality can be achieved through various techniques. Considering the essential role of vitamin D in the metabolism of calcium and phosphorus, reproduction in laying hens and eggshell quality (Mattila et al., 2003; Warren et al., 2020), the application of different sources of this vitamin (Cholecalciferol (vitamin D_3_); 25-hydroxycholecalciferol (25OHD_3_); 1,25- dihydroxycholecalciferol (1,25OH_2_D_3_); 1α-hydroxycholecalciferol (1αOHD3)) may provide beneficial effects.

Most research on the effects of different sources of vitamin D has been conducted on broiler chickens rather than on laying hens. Moreover, in the laying hens, researches have largely focused on the 25OHD_3_ and 1,25OH_2_D_3_ (Käppeli et al., 2011; Nascimento et al., 2014; Geng et al., 2018; Wang et al., 2020; Chen et al., 2020a; b; Lim & Ryu 2022; Jing et al, 2022; Li et al., 2023; Wang et al., 2023; Gao et al., 2024), while only a single study has investigated the effects of 1αOHD_3_ (Frost & Roland 1990). Moreover, it is critical to note that the efficiency of the kidneys in converting 25OHD_3_ to 1,25OH_2_D_3_ decreases in aged laying hens (Abe et al., 1982). This decline suggests that dietary inclusion of 1αOHD3, which is converted to 1,25OH_2_D_3_ in the liver, may be more advantageous than providing 25OHD_3_ in the late phase of production.

1αOHD_3_ serves as a synthetic analogue of 1,25OH_2_D_3_ (the hormonal form of vitamin D), characterized by the absence of a hydroxyl group on the 25th carbon. The molecule is simpler to chemically synthesis than of 1,25OH_2_D_3_ while its pattern of action is virtually identical (Haussler et al., 1973). 1αOHD_3_ offers a more cost-effective alternative for synthesis and dietary supplementation compared to 1,25OH_2_D_3_ (Haussler et al., 1973; Edwards et al., 2002) and, 1αOHD_3_ skips the important regulatory hydroxylation step normally mediated by 1α-hydroxylase in the kidney (Haussler et al., 1973; Miller & Portale, 2000). Following its absorption from dietary sources, the 1αOHD_3_ is subjected to hydroxylation in the liver at the 25th carbon, to become 1,25OH_2_D_3_ (Haussler et al., 1973). Studies comparing the biological activities of 25OHD_3_, 1,25OH_2_D_3,_ and 1αOHD_3_ in broilers have shown that while all metabolites exhibit vitamin D activity, 1αOHD_3_ which is hydroxylated in the liver demonstrates superior efficacy than 25OHD_3_ which is hydroxylated in the kidney in various physiological responses. Biopotency of the vitamin D metabolites decreases in the order: 1,25OH_2_D_3 =_ 1αOHD_3_ > 25OHD_3_ > vitamin D_3_ (Edwards, 2002; Edwards et al., 2002; Han et al., 2017).

Yang et al. (2020) conducted three experiments and found that broiler chickens exhibited optimal performance when their vitamin D_3_-free diets were supplemented with 2–5 μg/kg of 1αOHD_3_ from 1 to 42 days of age. According to a study by Hsiao et al. (2018), supplementing broiler chickens' diets with 1αOHD3, 25OHD_3_ or 1,25OH_2_D_3_ tended to improve FCR, bone mineral deposition, and bone-breaking strength. The results also showed that these vitamin D3 metabolites significantly upregulated the mRNA levels of genes involved in calcium and phosphorus homeostasis, such as Calbindin (calcium-binding protein), TRPV6 (transient receptor potential cation channel, subfamily V, member 6), β-glucuronidase, and Na/PiIIb. The addition of different levels of 1αOHD_3_ (0, 0.75, 1.5, 3, and 4.5 μg/kg) to the diets of laying hens did not significantly affect egg production, FI, FCR, egg quality, and eggshell strength. However, when 4.5 μg/kg of 1αOHD_3_ was included, there was a linear increase in the tibia weight, as well as serum phosphorus levels (Frost & Roland, 1990). Previous research showed that supplementing laying hen diets with 50 μg/kg 25OHD_3_ (basal diet containing 2000 IU/kg vitamin D_3_) from 55 to 63 weeks increased egg production, eggshell strength, Haugh unit, villus height (VH) to crypt depth (CD) ratio (VCR), tibia quality (including fresh tibia weight, strength, calcium content, and trabeculae area), serum alanine aminotransferase (ALT), calcium, and phosphorus antioxidant enzyme activities, while the content of total bilirubin, MDA, and alkaline phosphatase (ALP) in the serum decreased significantly (Gao et al., 2024).

As previously mentioned, to the extent of our knowledge, research investigating the effects of 1αOHD_3_ in laying hens has been extremely limited, with only one study conducted on this topic so far. Thus, the present study was conducted to evaluate the effects of different levels of 1αOHD_3_ on performance, eggshell quality, bone parameters, serum indexes, and morphology of spent laying hens.

## Material and methods

### Experimental design, animals, and diet

One hundred and eighty 110-week-old laying hens (LSL-Lite) were randomly divided into 5 groups with 6 replicates of six birds each (3 birds/cage, 44 × 40 × 40 cm) according to a completely randomized design. Each treatment had six replicates containing six hens each. The control group was fed a basal diet supplemented with 2000 IU/kg vitamin D. Four levels of 1αOHD_3_ (1.5, 3, 4.5, and 6 μg/kg) were added to the basal diet.

The commercial product Navashell®, containing 1αOHD_3_ (C_27_H_44_O_2_ - Vitamin Derivatives Inc, Athens, GA, USA), was purchased from Navanik (Tehran-Iran). Bird weight, production performance, egg weight, and egg quality were monitored before the experiment for 7 days to ensure that the replicates were distributed correctly. Before starting the experiment, the average egg mass and abnormal egg percentages for treatments were as follows: 0 μg/kg of 1αOHD_3_ (53.01 g, 2.41%), 1.5 of μg/kg 1αOHD_3_ (53.12 g, 2.44%), 3 of μg/kg 1αOHD_3_ (52. , 2.36%), 4.5 of μg/kg 1αOHD_3_ (53.18 g, 2.51%), and 6 of μg/kg 1αOHD_3_ (52.91 g, 2.49%). The results of the analysis of variance indicated that there were no significant differences between the treatments. After that, there was a one-week preliminary feeding period as an acclimation period. All birds were placed in cages and kept under 16 h light and 8 h dark for eight weeks. A corn-soybean meal basal diet was formulated to meet LSL-Lite strain nutrient requirements. The composition of dietary treatments and their nutrient contents are presented in Table 1.

**Table 1:** Ingredients and chemical composition of the basal diet (110–117 weeks of age).

Item (%)	Basal diet	Calculated composition	
Corn	58.32	Metabolizable energy(Kcal/Kg)	2700
Soybean meal	24.00	Crude protein (%)	15.60
Barley	2.00	Digestible amino acids (%)	
Soybean oil	1.67	Lys	0.70
Wheat bran	1.84	Met	0.40
DL-Methionine	0.20	Met + Cys	0.63
Di-calcium phosphate	1.24	Thr	0.49
Limestone	9.63	Na	0.15
Salt	0.18	Calcium (%)	3.93
NaHCO3	0.22	Available phosphorus (%)	0.30
Vitamin & Mineral premix^1^	0.60		
Toxin binder	0.10		

1. Vitamin and mineral premix provided (units per kg/feed): VA 9000 IU, VD3 2000 IU, VE 20 IU, VK 3 mg, VB1 1 mg, VB2 6 mg, VB6 5 mg, VB12 20 mcg, calcium pantothenate 30 mg, nicotinic acid 26 mg, biotin 38 mcg, folic acid 0.4 mg, choline 400 mg, iron, 25 mg; iodine, 0.45 mg; manganese, 80 mg; zinc, 60 mg; selenium, 0.2 mg.

### Production performance, egg quality and economic considerations

Egg production, egg weight, and abnormal eggs were recorded daily by replicate. Egg mass was calculated daily by multiplying egg production by egg weight. Feed was supplied to the birds two times per day, and FI was calculated by weighing the remaining feed at the end of each week. The FI and FCR (kg feed consumed per kg egg produced) were determined at one-week interval and for the entire experimental period. Any mortality was recorded and weighed as it occurred. Egg production and FI were adjusted for hen mortality. The abnormal egg proportion was calculated by dividing the total number of abnormal eggs (shell less, soft shell, cracked eggs) by the total number of eggs in each replicate.

At the 117th week of age, 25 eggs per treatment were randomly collected for egg quality analysis on the day of collection. Egg weight, eggshell weight, albumen weight, and yolk weight were measured using an electronic balance to the nearest 0.01 g and expressed as a percentage of the egg weight. Eggshell thickness was measured at three points using a micrometer gauge (VIJAY SURGICAL Micrometer Delhi, India) as described by Bozkurt et al. (2012). The Haugh unit (HU) was calculated using the following formula proposed by Haugh (1937).

$$\text{HU}\;\left(\%\right)\;=\;100\;\times \;\text{log}\;\left(H+7.57-1.7W^{0.37}\right)$$

where H is the height of the albumen in mm and W is the weight of the egg in g.

Economic performance was assessed by calculating the income over feed cost, which was derived from the revenue generated from egg sales and the cost of feed consumed by the hens. The gross revenue was determined by subtracting the feed cost from the selling price of all eggs. It is important to note that fixed costs related to the experiment, such as the initial purchase price of hens, veterinary care, housing, and labor, were not included in the analysis as they were consistent across all treatment groups (Elsherbeni et al., 2024).

### Serum biochemical parameters

At the end of the study, one bird from each replicate (as close as possible to the mean body weight) was randomly selected (6 birds per treatment) and slaughtered by severing the jugular vein. Blood samples were collected and centrifuged at 3,000 × g for 15 min within six hours of collection. The separated serum was then stored at -20 °C for further analysis. The serum levels of albumin, total protein, Aspartate aminotransferase (AST, Pars Azmoon Co., Tehran, Iran), ALT (Pars Azmoon Co., Tehran, Iran), ALP (Pars Azmoon Co., Tehran, Iran), calcium and phosphorus (Delta Darman Part, Tehran, Iran) as well as vitamin D (Monobind Inc-USA) were measured spectrophotometrically (UNICO-S2150UV- USA) using commercial kits following the manufacturer's instructions.

### Tibial quality analysis

One bird per replicate was selected and killed by cervical dislocation. The left (chemical analysis) and right (bone strength) tibia were removed, dissected, and cleaned of any adhering tissue. Tibia samples were dried at 105 °C for 24 h, then ashed at 550 °C for 24 h. Ash content was calculated as a percentage of dry-defatted weight. Ash from each tibia sample was processed for calcium analysis in accordance with the Association of Official Agricultural Chemists methodology (AOAC, 2005) in Bonyanndanesh Vet and Feed Analysis laboratory. The AOAC method for analyzing calcium in poultry bones typically involves a systematic approach that includes ashing, acid digestion, and subsequent quantification through titration. Concentrations of phosphorus in tibia ash were determined by the spectrophotometric method as described by Rutherfurd et al. (2004). The tibia diameter was measured at three points, one cm below the distal and proximal heads, and at the midpoint of the bone. The average of these measurements was calculated. The tibia breaking strength at the midpoint along its length was determined with an automated testing machine (Universal Testing Machine, STM-20 Cap. 20 KN, SANTAM Co. Tehran, Iran).

### Intestinal morphology

At the end of study, six birds per treatment (closer to the mean body weight for each replicate) were selected, killed via cervical dislocation. Immediately after slaughter, two-cm section of the jejunum (5 cm anterior to the Meckel's diverticulum) was excised, fixed in 10% neutral buffered formalin, processed in graded alcohol series and then cleared in xylene. The samples were embedded in paraffin and cut using a CUT4055 manually microtome (microTec Laborgeräte GmbH, Germany). Histological sections were stained with hematoxylin-eosin and digitalized with a computer-aided light microscope (Olympus BX51TF, Japan). The histological parameters recorded included villus height (VH), villus width (VW), at the half height point of villus, crypt depth (CD), and villus height to crypt depth ratio (VCR). Villus surface area (VSA) was calculated using a formula described by Sakamoto et al. (2000).

VSA=2π×(VW/2)×VH,

where. π= 3.14, VW=villus width in μm and VH=villus height in μm.

### Statistical analysis

The orthogonal polynomial contrast test was performed to determine linear and quadratic effects of increasing inclusion level of 1αOHD_3_ in diets on each measurement. After visualizing the data, four independent regression models, including linear, quadratic, linear plateau, quadratic plateau were fit to the data using the *easynls* package (Arnhold 2017) in R Core Team (2023). We compared the fit of these models using Akaike's Information Criterion (Burnham and Anderson 2003).

## Results

### Production performance, egg quality and economic considerations

Dietary feeding of incremental levels of 1αOHD_3_ increased the egg production (linear P = 0.002 and quadratic P = 0.009) whilst it decreased the abnormal eggs (linear P = 0.004 and quadratic P = 0.009) in aged laying hens (Table 2). In addition, the linear tendency to increase was observed in egg mass (P = 0.075) with increasing dietary 1αOHD_3_ supplementation levels. However, egg weight, FI, and FCR showed neither a linear nor a quadratic effect (P > 0.05). As shown in Table 2, total income (linear P = 0.008 and quadratic = 0.031), feed cost (linear P = 0.009 and quadratic = 0.035), and gross revenue (linear P = 0.042) increased with increasing inclusion of 1αOHD_3_. The addition of 1αOHD_3_ to the basal diet had no significant effect on egg quality parameters (Table 3) including Haugh units, the relative weight of yolk, albumen, and eggshell (P > 0.05). Linear and quadratic responses were found for shell thickness and shell strength (P < 0.001).

**Table 2:** Effects of dietary levels of 1αOHD_3_ on production performance, abnormal eggs and economic efficiency of laying hens from 110 to 117 weeks of age.

	1αOHD_3_ levels (μg/kg)					SEM	P-value	
	0	1.5	3	4.5	6		Linear	Quadratic
Egg production (H.D. %)	79.8	80.3	80.7	82.3	82.3	0.632	0.002	0.009
Egg weight (g)	63.0	63.1	63.1	63.8	63.4	0.731	0.575	0.851
Egg mass (g)	50.4	50.5	50.8	52.5	51.7	0.679	0.075	0.211
Feed intake (g/hen/day)	117.6	115.9	117.2	116.8	115.7	0.624	0.250	0.504
FCR	2.35	2.32	2.37	2.27	2.25	0.041	0.111	0.236
Abnormal eggs (%)	3.30	3.06	3.23	2.82	2.55	0.204	0.004	0.009
Total income/hen/day (Rial) *	26493	26776	26896	27868	27775	20.651	0.008	0.031
Feed cost/hen/day (Rial) *	16585	16485	16813	16894	16866	5.554	0.009	0.035
Gross revenue/hen/day (Rial) *	9908	10291	10084	10974	10909	19.180	0.042	0.133

A significant difference was considered with *P* < 0.05. A tendency toward difference was considered with 0.05 < *P* < 0.10.

⁎ The percentage of abnormal eggs has been considered in the calculation of the gross revenue.

**Table 3:** Effects of dietary levels of 1αOHD_3_ on egg quality parameters in laying hens at the end of experiment (117 weeks).

	1αOHD_3_ levels (μg/kg)					SEM	P-value	
	0	1.5	3	4.5	6		Linear	Quadratic
Shell weight (%)	10.08	10.01	9.76	10.11	10.03	0.278	0.994	0.865
Albumin weight (%)	55.7	56.1	56.1	56.0	56.4	0.532	0.378	0.678
Yolk weight (%)	28.37	28.44	28.36	28.16	28.08	0.319	0.338	0.593
Haugh unit	70.4	72.5	71.6	69.8	69.1	2.268	0.426	0.514
Shell thickness (mm)	445.8	452.2	458.0	479.7	479.0	6.294	<0.001	<0.001
Eggshell strength (N)	36.8	37.0	37.1	38.1	38.3	0.331	<0.001	<0.001

A significant difference was considered with *P* < 0.05. A tendency toward difference was considered with 0.05 < *P* < 0.10.

### Serum biochemical parameters

Data describing the effect of 1αOHD_3_ supplementation on serum metabolites and enzymes activities are presented in Table 4. The serum concentrations of calcium (linear P = 0.003 and quadratic P = 0.011), albumin (linear P = 0.016 and quadratic P = 0.033), vitamin D (linear and quadratic P < 0.001) and ALT activity (linear P = 0.02) were increased with 1αOHD_3_ supplementation in aged laying hens. ALP activity decreased linearly (P = 0.003) and quadratically (P = 0.011) with increasing 1αOHD_3_. Level of 1αOHD_3_ did not affect P and AST activity in the serum of aged laying hens (P > 0.05). The linear plateau showed that the optimal serum albumin levels might be achived with 4.27 μg 1αOHD_3_/kg of diet.

**Table 4:** Effects of dietary levels of 1αOHD_3_ on serum biochemical indicators of laying hens at the end of experiment (117 weeks).

	1αOHD_3_ levels (μg/kg)					SEM	P-value	
	0	1.5	3	4.5	6		Linear	Quadratic
Ca (mg/dl)	18.70	19.08	19.11	20.13	19.88	0.372	0.003	0.011
P (mg/dl)	7.10	7.47	7.27	8.22	8.27	0.611	0.109	0.276
Albumin (g/dl)	1.95	2.37	2.28	2.53	2.48	0.173	0.016	0.033
Total Protein (g/dl)	6.23	6.36	6.27	6.68	6.75	0.229	0.062	0.163
Vitamin D (ng/ml)	63.1	66.3	65.0	71.5	71.0	1.826	<0.001	<0.001
ALP (U/L)	320.2	295.0	306.3	281.5	284.0	9.563	0.003	0.011
AST (U/L)	64.3	66.5	65.2	67.0	68.3	3.721	0.481	0.780
ALT (U/L)	33.7	34.5	35.3	36.8	37.5	1.384	0.020	0.069

A significant difference was considered with *P* < 0.05. A tendency toward difference was considered with 0.05 < *P* < 0.10.

ALP: Alkaline phosphatase; AST: Aspartate aminotransferase; ALT; Alanine aminotransferase.

### Tibial quality analysis

As shown in Table 5, tibia diameter (linear P = 0.053), tibia strength (linear and quadratic P < 0.001) and calcium percentage of the tibia (linear P = 0.004 and quadratic P = 0.014) increased with increasing inclusion of 1αOHD_3_. No difference in ash and phosphorus percentage of the tibia was observed among all treatments (P > 0.05). According to quadratic plateau model, the optimum levels of 1αOHD_3_ for maximizing tibia dimeter would be 2.44 μg/kg.

**Table 5:** Effects of dietary levels of 1αOHD_3_ on tibia quality in laying hens at the end of experiment (117 weeks).

	1αOHD_3_ levels (μg/kg)					SEM	P-value	
	0	1.5	3	4.5	6		Linear	Quadratic
Tibia diameter (mm)	6.74	7.29	7.37	7.26	7.53	0.251	0.053	0.114
Ash (%)	50.3	51.0	50.5	51.1	51.6	0.724	0.247	0.500
Ca (% of tibia weight)	21.43	21.67	22.87	22.70	23.29	0.538	0.004	0.014
P (% of tibia weight)	10.66	10.16	10.19	9.90	10.35	0.342	0.429	0.346
Tibia strength (N)	111.7	113.1	111.2	127.7	132.6	4.501	<0.001	<0.001

A significant difference was considered with *P* < 0.05. A tendency toward difference was considered with 0.05 < *P* < 0.10.

### Intestinal morphology

The effect of dietary 1αOHD_3_ incorporation on the intestinal morphology of aged laying hens is shown in Table 6. Linear and quadratic responses were found for CD (linear and quadratic P = 0.001) and VCR (linear P = 0.004 and quadratic P = 0.010). The results showed that dietary inclusion of 1αOHD_3_ induced no linear or quadratic effects (P > 0.05) on jejunal morphology of aged laying hens including VH, VW and VSA. Linear and quadratic plateau models indicated that the minimum CD might be obtained with 4.43 μg/kg of 1αOHD_3_, whereas the maximum VH/CD might be achieved with 4.45 μg/kg of 1αOHD_3_

**Table 6:** Effects of dietary levels of 1αOHD_3_ on the intestinal morphology in laying hens at the end of experiment (117 weeks).

	1αOHD_3_ levels (μg/kg)					SEM	P-value	
	0	1.5	3	4.5	6		Linear	Quadratic
Villus height (μm)	1056.2	1076.4	1039.2	1087.8	1080.4	38.641	0.633	0.875
Villus width (μm)	202.8	204.9	219.9	216.3	206.4	12.132	0.633	0.587
Villus surface area (mm^2^)	0.680	0.696	0.721	0.724	0.698	0.323	0.641	0.725
Crypt depth (μm)	183.1	167.0	158.1	155.9	153.8	7.022	0.001	0.001
Villus height/crypt depth	5.86	6.45	6.58	7.04	6.97	0.040	0.004	0.010

A significant difference was considered with *P* < 0.05. A tendency toward difference was considered with 0.05 < *P* < 0.10.

## Discussion

Maintaining high laying rates, eggshell quality, and bone health are major concerns for the poultry industry when managing laying hens towards the end of their production cycle. Earlier studies have indicated that the laying rate and egg quality, such as eggshell strength and thickness, deteriorate with age in hens. High egg production rates in laying hens can lead to avian osteoporosis, primarily due to the resorption of both structural and medullary bone to meet the demands of eggshell formation in the later stages of their production cycle (Whitehead & Fleming, 2000). Nutritional strategies have the potential to alleviate the negative effects of aging on production performance, bone health, and egg quality in laying hens at the late-laying phase (Gao et al., 2024). As previously highlighted, the research on the effects of 1αOHD_3_ on laying hens is quite limited and the biological potency of 1αOHD_3_ is comparable to that of 1,25OH_2_D_3_ and higher than that of cholecalciferol and 25OHD_3_ (Edwards, 2002; Edwards et al., 2002; Han et al., 2017). Therefore, this study focuses on evaluating the influence of adding 1αOHD_3_ to laying hen diets containing 2000 IU/Kg vitamin D on performance, serum parameters, tibia quality, and jejunal morphology. Given the scarcity of studies focused on 1αOHD_3_ supplementation in laying hens, the discussion necessarily incorporates findings from studies that supplemented other vitamin D metabolites (25OHD_3_ or 1,25OH_2_D_3_) in laying hens or 1αOHD_3_ in broiler diets.

Our results showed that linear and quadratic effects in the egg production and there was a trend of linear effect for egg mass. However, FI, egg weight, and FCR were not affected by dietary treatment. These findings are consistent with previous research by Chen et al. (2020a), Jing et al. (2022), Lim and Ryu (2022), and Gao et al. (2024). Previous studies confirm that 25OHD_3_ improves egg production in laying hens under 62 weeks of age, without affecting FI, egg weight, and FCR. However, these benefits are not seen in hens aged 62–95 weeks. (Chen et al., 2020a; Lim & Ryu, 2022). The lack of effect of 25OHD_3_ supplementation on egg production in aged laying hens may be associated with a decrease in kidney efficiency for converting 25OHD_3_ to 1,25OH_2_D_3_. The positive effects of dietary 1αOHD_3_ supplementation on egg production may be attributed to an increase in VSA (numerically) in the jejunum in our study, which enhances nutrient absorption from the diet. Additionally, our findings indicate that supplementing the diet of laying hens with 1αOHD_3_ led to a linear and quadratic increase in the serum levels of vitamin D and it has been previously confirmed that the supplementation of vitamin D_3_ in laying hen diets led to elevated levels of FSH and LH, indicating that sufficient dietary vitamin D_3_ may enhance the feedback regulation of hormone secretion from the hypothalamus and pituitary gland, which in turn supports follicle development (Geng et al., 2018). Our findings contrast with those of several studies that reported no significant effects of vitamin D metabolites (25OHD_3_, vitamin D_3_, vitamin D_2_) supplementation on egg production in laying hens (Frost & Roland, 1990; Käppeli et al., 2011; Adhikari et al., 2020; Li et al., 2023; Warren et al., 2024). The discrepancies observed in the results of various studies may be attributed to differences in the form and level of vitamin D metabolites, dietary calcium levels, the age of the laying hens, level of vitamin D in the basal diet, and duration of the experiment. The present study demonstrates that broken egg rate decreased from 3.30 to 2.55 percent, while shell thickness and strength increased from 445.81 to 479.02 mm and from 36.84 to 38.34 N, respectively as the amount of 1αOHD_3_ in the diets increased. Our study demonstrates an increase in egg production profitability due to the inclusion of 1αOHD_3_ in the diet. While this profit may seem modest, it can be quite substantial when applied to large-scale egg production. Notably, no studies have yet examined the impact of vitamin D metabolites on egg production profitability. These findings are consistent with previous research by Koreleski, and Witkiewicz (2005), Geng et al. (2018), Wen et al. (2019), Jing et al. (2022), Wang et al. (2020, 2023), Gao et al. (2024) who observed that 25OHD_3_ or vitamin D_3_ supplemented diets increased the eggshell thickness and strength and decreased abnormal eggs, but Frost and Roland (1990), Chen et al. (2020), Lim and Ryu (2022), Li et al. (2023) stated that the addition of 1αOHD_3_ or 25OHD_3_ to the diet of laying hens had no significant effect on eggshell thickness and strength. The reduction in eggshell strength and thickness is a common phenomenon observed in the spent layers (Qiu et al., 2020; Park & Sohn, 2018). The increase in eggshell thickness and strength observed in our study may be attributed to the increased palisade layer thickness, along with a higher density of mammillary knobs and a notable reduction in mammillary knob width (Jing et al., 2022), resulting in fewer abnormal eggs. An additional factor likely influencing the increased thickness and strength of the eggshell in our study is the higher serum levels of calcium and vitamin D observed in birds that received 1αOHD_3_. Vitamin D is known to regulate calcium and phosphorus metabolism, which are critical for bone development and eggshell development (Fleming 2008). Furthermore, it enhances endometrial morphology (Jing et al., 2022) and increases the expression of the mRNA levels of genes like Calbindin, TRPV6, β-glucuronidase, Na/PiIIb, and vitamin D receptors that play a role in calcium and phosphorus homeostasis (Van Cromphaut et al., 2001; Fleming 2008; Hsiao et al., 2018; Yang et al., 2020). These results may underscore the increasing importance of vitamin D and its metabolites in egg production and eggshell quality, particularly in aged laying hens and suggest that a level of 2000 IU/kg vitamin D may not be sufficient to achieve the genetic potential in late period laying hens.

Consistent with our observations, previous researches has also demonstrated that supplementing the diets of broiler chickens with 1αOHD_3_ (Han et al., 2013; 2018; Warren et al., 2020; Zhang et al., 2020) and laying hens with 25OHD_3_ and vitamin D (Adhikari, 2020; Lim and Ryu 2022; Gao et al., 2024) resulted in similar improvements in serum calcium but had no effect on serum phosphorus content (Zhang et al., 2020; Lim & Ryu 2022). In contrast to our findings, Nascimento et al. (2014) in laying hens and Hsiao et al. (2018) in broiler chicks reported that various sources of vitamin D did not influence serum calcium levels. The conflicting results observed in various studies could be due to the bird type (broiler or layer), form and level of vitamin D metabolites, the timing of sample collection, and the vitamin D concentrations in the diet of the control group. Vitamin D is vital for blood calcium homeostasis, as it regulates calcium absorption in the intestines, enhances reabsorption in the kidneys, and plays a key role in both bone mineralization and mobilization (Christakos et al., 2017; Goltzman et al., 2024). 1αOHD_3_ is hydroxylated by 25 hydroxylase in the liver to 1,25OH_2_D_3_, known as calcitriol, and recognized for its ability to enhance the absorption of calcium and phosphorus from the diet through upregulation of vitamin D receptor (Fleming 2008; Han et al., 2018; Warren et al., 2020). The vitamin D receptor plays a critical role in the signal transduction of genes involved in calcium (calbindin and TRPV6) and phosphorus (NaPiIIb) absorption when 1,25OH_2_D_3_ binds to it (Van Cromphaut et al., 2001). 1,25OH_2_D_3_ has the potential to regulate the expression of ca-transport related proteins such as carbonic anhydrase in the eggshell gland, influencing the biochemical processes involved in eggshell formation (Bar 2008). These actions is fundamental to maintaining optimal mineral levels in the body, which are necessary for numerous biological processes. Researches had shown that the administration of 1αOHD_3_ significantly elevates the vitamin D receptor protein levels in the intestinal of broiler chicks (Han et al., 2018; Yang et al., 2020), subsequently resulting in higher mRNA expression of NaPiIIb in the duodenum, jejunum, and ileum of broilers (Han et al., 2009; Yang et al., 2020). The NaPiIIb protein is the transporter of phosphate in the small intestine. Despite the lack of effect of the experimental treatments on serum phosphorus levels in our study, serum phosphorus levels in the 4.5 or 6 μg/kg of 1αOHD3 groups were approximately 16% higher than those in the control group. As shown in our experiment, vitamin D in the serum of laying hens increased linearly and quadratically with 1αOHD3, which may explain the observed effects on serum calcium and phosphorus levels. Our research revealed that dietary supplementation with 1αOHD_3_ in laying hens resulted in higher serum levels of vitamin D. Previous studies have demonstrated that dietary supplementation with 25OHD_3_ significantly increases its circulating levels in the blood and promotes the development of bone structure, enhancing mineral deposition and bone quality during the laying period (Chen et al., 2020b; Wang et al., 2020; Zhang et al., 2020; Liermann et al., 2023). In contrast to our findings, Warren et al. (2020) observed no significant influence of dietary addition of 5 µg/kg 1αOHD_3_ in the diet of broiler chickens on the serum vitamin D concentration. The mechanism by which 1αOHD_3_ influences serum vitamin D3 levels remains unclear.

Our findings indicate that the supplementation of 1αOHD_3_ to the diets of laying hens reduced ALP activity and increased ALT activity linearly and quadratically, while AST activity was unaffected by dietary treatments. In laying hens, ALP activity is often linked to deposition of calcium and phosphorus, skeletal mineralization, and osteoblastic activity, which is critical for maintaining bone health, especially during egg production. In a study by Gao et al. (2024), the dietary addition of 50 μg/kg 25OHD_3_ + 2000 IU vitamin D3 increased serum ALT activity and decreased ALP activity compared to the control group (containing 4000 IU vitamin D3), while no remarkable differences in the activity of AST were observed among the dietary groups. A reduction in ALP activity in our study could be linked to the presence of circulating vitamin D, which plays a crucial role in calcium metabolism and utilization. This relationship may help mitigate bone disorders that are associated with elevated ALP activity. The numerical increase in serum albumin levels observed in the treatments supplemented with 1αOHD_3_ in our experiment could facilitate the efficient transport of minerals and hormones, which may positively influence the physiology of laying hens.

In the present study, the tibia diameter increased linearly with 1αOHD_3_, while tibia Ca and tibia strength increased linearly and quadratically with 1αOHD_3_ supplementation. The findings of this study are in agreement with those of Chen et al. (2020), Wang et al. (2020), Zhang et al. (2020), Li et al. (2023), Gao et al. (2024), in which adding different sources of vitamin D3 to the diets of laying hens and broilers increased the tibia weight, strength, and calcium content, although no such effect was observed at earlier ages (Li et al., 2023). The results of a recent study demonstrate that the inclusion of 25OHD3 in the diets of pullets has significant potential in promoting the development and maintenance of healthy tibia in laying hens (Li et al., 2021). The findings of Han et al. (2018) in broilers, indicated that the weight, ash weight, and both ash and calcium percentages of the bone in birds receiving the 1αOHD_3_ diet were significantly lower than those in the control group, while increasing dietary 1αOHD_3_ levels significantly improved tibia mineralization and the retention of calcium and total phosphorus. In our study, the control diet contained 2000 IU/kg vitamin D3, and different levels of 1αOHD_3_ were added to this diet. The calcium and phosphorus levels were consistent across all treatments. In contrast, in the study by Han et al. (2018) the basal diet, to which different levels of 1αOHD_3_ were added, did not contain vitamin D3 (the control diet contained 25 μg/kg of D3), and the calcium and phosphorus levels in basal diet were reduced by half compared to the control diet. These dietary variations may account for the differences observed in the results between the two studies. Calcium and phosphorus homeostasis in the body is regulated by a balance between intestinal absorption, renal excretion, bone metabolism and eggshell calcium deposition. As explained earlier, the efficiency of the kidneys in converting 25OHD_3_ to 1,25OH_2_D_3_ decreases in aged laying hens (Abe et al., 1982), this decline may be compensated by 1αOHD3, which can be converted to 1,25OH_2_D_3_ in the liver, potentially leading to increased levels of vitamin D and 1,25OH_2_D_3_ in the body. The 1,25OH_2_D_3_ enhances mineral homeostasis through upregulation of mRNA levels of genes involved in calcium and phosphorus absorption such as vitamin D receptors, TRPV6, calbindin, NaPiIIb (Van Cromphaut et al., 2001; Fleming 2008; Hsiao et al., 2018; Han et al., 2018; Warren et al., 2020), as well as the elevation of calcium channels and transport proteins in the intestine and kidney (Cai et al., 1993), thereby improving bone parameters, particularly bone strength in our study (Theofan et al., 1987; Meyer et al., 1992; Cai et al., 1993).

Another reason that could explain the increased strength of the tibia is likely due to an enhancement in the arrangement of bone trabeculae tightly, the number and the area of trabeculae. This enhancement, leads to improved bone trabecular structure and bone connectivity, ultimately contributing to increased bone resistance to fractures (Chen et al., 2020; Wang et al., 2020; Gao et al., 2024). As a result, dietary supplementation of 1αOHD_3_ can improve bone resistance to fractures in aged laying hens, contributing to a reduction in the incidence of bone disorders.

This study revealed that dietary supplementation of 1αOHD_3_ in laying hens caused a linear and quadratic decrease in the CD and an enhancement in the VCR, while it had no significant effect on VH, VW and VSA Some studies have demonstrated that dietary supplementation with different vitamin D sources can enhance intestinal morphology (Geng et al., 2018; Wang et al., 2023; Suarez et al., 2023; Gao et al., 2024). Gao et al. (2024) concluded that dietary supplementation of 25OHD_3_ in laying hens resulted in increased VH and VCR, as well as a reduction in crypt depth. A recent study by Wang et al. (2023) found that the dietary supplementation of 25OHD_3_ in laying hens for 8 weeks under *Escherichia coli* lipopolysaccharide challenge improved intestinal morphology (higher VCR and lower CD) in the duodenum and jejunum, compared to a control group. A possible explanation for the differences in our findings compared to other studies may be the type of birds (broiler in Suarez et al., 2023), sources of vitamin D (25OHD_3_ in other studies), and stress challenges such as disease (*Escherichia coli* lipopolysaccharide challenge in Wang et al., 2023 and coccidiosis vaccine in Suarez et al., 2023). While aging in laying hens is associated with a reduction in the functional role of intestinal morphology, nutritional interventions can serve to ameliorate these effects. The reduced crypt depth observed in birds fed diets supplemented with 1αOHD_3_ may suggest a higher presence of mature enterocytes within the villi, indicating decreased cell recruitment and, consequently, lower energy expenditure required for maintaining absorptive function, while deeper crypts are indicative of villus atrophy in the small intestinal mucosa, which correlates with a reduced absorptive capacity.

## Conclusion

In summary, the results of this study indicated that the addition of 1αOHD_3_ to the diets of aged laying hens enhances gastrointestinal structure and positively impacts serum concentrations of calcium, albumin, total protein, vitamin D, and key enzymes such as ALP and ALT. These improvements lead to enhanced egg production, increased eggshell thickness and strength, and a reduction in abnormal eggs and consequently, this has resulted in greater profitability for egg production. However, further research is necessary to elucidate how 1αOHD_3_ influences these parameters and the associated metabolic pathways. Also, 1αOHD_3_ has contributed to an increase in bone strength, tibia diameter and calcium content in the tibia, which is very important for spent laying hens that suffer from a decline in their bone quality parameters.

## Declaration of competing interest

The authors declare no conflicts of interest.
